# Thermal behaviour of *Anopheles stephensi *in response to infection with malaria and fungal entomopathogens

**DOI:** 10.1186/1475-2875-8-72

**Published:** 2009-04-20

**Authors:** Simon Blanford, Andrew F Read, Matthew B Thomas

**Affiliations:** 1 School of Biological Sciences, University of Edinburgh, Edinburgh, Scotland, UK; 2 Centre for Infectious Disease Dynamics, Departments of Biology and Entomology, The Pennsylvania State University, University Park, Pennsylvania 16802, USA

## Abstract

**Background:**

Temperature is a critical determinant of the development of malaria parasites in mosquitoes, and hence the geographic distribution of malaria risk, but little is known about the thermal preferences of *Anopheles*. A number of other insects modify their thermal behaviour in response to infection. These alterations can be beneficial for the insect or for the infectious agent. Given current interest in developing fungal biopesticides for control of mosquitoes, *Anopheles stephensi *were examined to test whether mosquitoes showed thermally-mediated behaviour in response to infection with fungal entomopathogens and the rodent malaria, *Plasmodium yoelii*.

**Methods:**

Over two experiments, groups of *An. stephensi *were infected with one of three entomopathogenic fungi, and/or *P. yoelii*. Infected and uninfected mosquitoes were released on to a thermal gradient (14 – 38°C) for "snapshot" assessments of thermal preference during the first five days post-infection. Mosquito survival was monitored for eight days and, where appropriate, oocyst prevalence and intensity was assessed.

**Results and conclusion:**

Both infected and uninfected *An. stephensi *showed a non-random distribution on the gradient, indicating some capacity to behaviourally thermoregulate. However, chosen resting temperatures were not altered by any of the infections. There is thus no evidence that thermally-mediated behaviours play a role in determining malaria prevalence or that they will influence the performance of fungal biopesticides against adult *Anopheles*.

## Background

New strategies for the control of malaria and the vectors that transmit the disease continue to be sought. One possibility is indoor residual treatments of biopesticides containing spores of entomopathogenic fungi, which infect adult *Anopheles *on contact. Initial studies using this approach in lab and field settings have demonstrated clear potential for certain fungi to reduce malaria transmission capacity of *Anopheles *mosquitoes, through a combination of both lethal [1,2] and sub-lethal [1,3] effects.

The fungal biopesticide approach for malaria control follows an earlier programme of research that developed, tested and registered a fungal biopesticide for control of locusts and grasshoppers in Africa [see [4,5]]. This biopesticide product can provide effective control of a range of locust and grasshopper pests with minimal environmental impact [4,6-9]. However, its performance in the field has been shown to vary considerably depending on environmental temperature and the behavioural thermoregulation of infected insects [8-11]. Part of this variation derives from the direct effect of temperature on fungal growth [8,9,12,13], but behavioural fever, in which locusts increase body temperature by basking for longer or in warmer sites, is a key defence response that can dramatically affect speed of kill (virulence) following infection [10,14,15]. Fungal biopesticide control of locusts is generally good under environmental conditions that constrain thermoregulation, with the majority of infected locusts or grasshoppers dying by 7–14 days [16]. Conditions that enable locusts to attain behavioural fevers can, on the other hand, prolong survival for many weeks [14,17]. Hence, successful deployment of the biopesticide technology for locust and grasshopper control centres on an understanding of environmental conditions and host thermal behaviour [8,9]. An important question that follows, therefore, is whether susceptibility of adult mosquitoes to fungal pathogens will be similarly affected by anopheline thermal behaviour and accordingly, whether use of a fungal biopesticide for malaria control can be better informed and subsequently directed if any, potentially subtle, temperature mediated interactions are understood.

The influence of temperature on *Anopheles *is well understood in a general sense with, for example, aspects such as larval development time [18,19] and the thermal limits of adult distribution [20] relatively well characterized. However, studies allowing adult mosquitoes to make choices in response to temperature are very limited. Kirby and Lindsay showed that uninfected *Anopheles arabiensis and Anopheles gambiae *avoid temperatures above 33°C [21], but no other studies employing choice environments. The vast majority of studies exploring effects of temperature on mosquito and/or malaria life history subject the vector and/or parasite to constant temperature conditions. This is the case even though many ectothermic animals [e.g. [22-24]], including a range of insect species [10,25-28], often alter thermal behaviour following infection with a microbial agent (or extract thereof). The first objective of the studies reported here was to determine whether adult *Anopheles stephensi *exhibited a thermal preference on a simple thermal gradient and whether infection with various entomopathogenic fungi would alter thermal responses.

Temperature also affects the development of malaria parasites within mosquitoes [20,29-34]. Low environmental temperatures are thought to impact on sporogony by slowing or even halting development, though it is not clear whether low temperature impacts directly on the parasite or indirectly by retarding vector competence [20]. High environmental temperatures following an infective blood meal can inhibit early malaria development [32-34]. These restrictive temperatures, which are within the thermal tolerance range of the mosquitoes themselves [21], need not be sustained for a long to negatively influence malaria development [32]. It might, therefore, be advantageous for the malaria parasite to manipulate mosquito behaviour to avoid resting at high temperatures during this thermally sensitive phase. *Plasmodium *can manipulate the fecundity and blood feeding behaviour of its vector [see [35] and references therein] and, of particular relevance in a thermal context, the lizard malaria *Plasmodium mexicanum *has been shown to manipulate the thermal behaviour of its sand fly vector, with warmer resting sites preferred by infected flies, so increasing the rate of parasite development and hence transmission [36]. The second objective of this study was to examine whether malaria infection (in this case the rodent malaria, *Plasmodium yoelii*) affected the thermal behaviour of *An. stephensi*, potentially constraining selection to lower temperatures immediately following blood feed.

Note that if infected hosts have different thermal preferences, it can be very difficult to determine whether the changes are because the host is altering temperature to limit the virulence of a pathogen, or whether an infectious agent is manipulating host temperature to enhance its own fitness [37]. The study reported here investigates for *An. stephensi*, whether infection with malaria or candidate biopesticidal fungi alters thermal site selection in the first place.

## Methods

### Mosquito rearing

*Anopheles stephensi *was reared under standard insectary conditions at 26°C, 75% humidity and a 12 L:12 D photo-period. Eggs were placed in plastic trays (25 cm × 25 cm × 7 cm) filled with 1.5 l of distilled water. To reduce variation in adult size at emergence, larvae were reared at a fixed density of 400 per tray. Larvae were fed on Liquifry for five days and then on TetraFin fish flakes. From approximately two weeks after egg hatch, pupae were collected daily and placed in emergence cages. The adults that emerged were fed *ad libitum *on a 10% glucose solution supplemented with 0.05% paraaminobenzoic acid (PABA). The experiments used four to six day old adult female mosquitoes, that were distributed between experimental cages 48 hours prior to receiving a blood meal.

### Determination of thermal preference on a gradient

The general approach was to generate "snapshot" estimates of resting temperatures on thermal gradients, rather than monitor behaviour over a prolonged exposure, as this has been found adequate for showing altered thermal behaviour in other insect-pathogen and parasite studies [e.g. [11,38,39]]. Two thermal gradients were constructed, each comprising an aluminium sheet (5 mm thick) resting on a heating pad (15 cm × 28 cm, 7 Watt Habistat™ Heat Mat) at one end and an ice pack at the other. A sheet of chromatography paper marked at centimetre intervals to provide a gauge was placed on top of the aluminium. A perspex lid created six channels, 25 cm long, 2.0 cm wide and 1.0 cm deep. The channels were closed at the hot end and had a removable Perspex strip at the cold end to enable introduction of mosquitoes. In addition, the Perspex lid had frequent small holes drilled in it to assist air circulation and facilitate temperature measurement within the gradient. Prior to the experimental runs, the chromatography paper was lightly sprayed with distilled water to maintain humid conditions. When set up, temperature within each channel ranged from 14°C to 38°C increasing linearly along the surface of the gradient. Temperatures just beneath the lid (i.e. 1 cm above the gradient surface) increased in a similar but non-linear fashion. Hence, mosquitoes had slightly more opportunity to perch at temperatures in the middle range of those available on the gradient as they could rest on both the roof and the surface

To assess selection of thermal resting site, mosquitoes were transferred from their holding cages into an appropriate channel of a gradient using an aspirator. Mosquitoes were blown to the centre of the channel from the cold end. For each run, six mosquitoes of the same treatment were introduced into a single channel. Treatments were assigned to channels and gradients randomly for each exposure. After all lanes were filled, mosquitoes were disturbed by gently tapping the top of the gradient. The lights were then turned off and the mosquitoes allowed to settle for 30 minutes. Following this period red light was used to note the exact resting position of each insect on the gradient. After positions were recorded, the channels were flooded with CO_2 _and the insects removed to new cages appropriate to their treatment group (one cage per treatment) where they were provided with 10% glucose solution *ad libitum*. The pre- and post-exposure cages were maintained in the same insectary. All insects recovered from the exposure to CO_2 _within one minute of knockdown. The temperature of the resting position of each insect noted under the red light, was then measured with a fine diameter thermocouple attached to a fast response digital thermometer. The gradients were wiped clean with 70% ethanol, the marked chromatography paper changed and a fresh ice pack installed. The gradient temperature was allowed to settle and a further batch of insects introduced. 36 mosquitoes from each treatment were assessed per day, with individual mosquitoes exposed only once to the gradient during the course of the study.

### Experiment 1: Fungal isolates, blood feeds and exposure procedure

Three fungal isolates, a *Beauveria bassiana *isolate IMI391510 (hereafter *Beauveria*), a *Metarhizium anisopliae *var. *anisopliae *isolate F52 (hereafter F52) and a *M. anisopliae *var *acridum *isolate IMI330189 (hereafter M189) were used in the study. *Beauveria *was chosen as it has shown promise as a biocontrol agent against *An. stephensi *[1]. F52 was investigated because, in previous tests, it has shown an intermediate level of virulence (50 – 70% mortality in 14 days, authors' unpublished data). The *M. anisopliae *var *acridum *isolate has shown only very low virulence against *An. stephensi *(generally less than 10% mortality in 14 days, authors unpublished data) but is known to elicit behavioural fever responses in other insects [e.g. see [10,14]]. Thus, three distinct fungal strains were considered covering a range of virulence.

Each isolate was formulated in a mix of mineral oils [1] and the spore concentration adjusted to give 1 × 10^9 ^spores ml^-1^. Spray applications employed a hand held artist's air brush to produce an aerosol the spore formulation.

Female *An. stephensi *were taken from emergence cages and randomized into smaller experimental cages (20 × 20 × 20 cm) for mouse feeds. There were six cages for each isolate and a further six for control mosquitoes, which received a blood feed but were not exposed to the spray aerosol. Each cage contained approximately 100 mosquitoes and glucose solutions were removed 24 hours prior to feeds. Mice (c57BL/6J) were anaesthetized and one placed on top of each cage and the mosquitoes allowed to feed for 20 minutes. Following feeds all mosquitoes that had not taken a full blood meal were removed.

For each isolate 0.5 ml^-1 ^of the fungal spore formulation was sprayed evenly across four cage sides with the mosquitoes *in situ*. The mosquitoes were left for one hour following the spray application. This application procedure exposes mosquitoes to the spray residue on the mesh surface of the cage but also to direct contact with spray droplets. In this way mosquitoes were very unlikely to avoid contacting spores. Following the exposure period, sub-samples were taken from each cage and placed in three further cages (50 mosquitoes per cage, three cages per fungal isolate and control) to be monitored for survival as a check on spray efficacy. Remaining mosquitoes were removed and placed in large emergence cages (one per treatment) and housed in an insectary at 26°C and 75% relative humidity. These insects were used for gradient exposures from day 2 through to day 6 post spray treatment.

### Experiment 2: *Plasmodium yoelii *infections, gradient exposures and dissection procedures

Experimental mice (c57BL/6J) were infected with 10^6 ^parasites of the rodent malaria, *P. yoelii *(clone 33X, from the World Health Organization Registry of Standard Malaria Parasites, University of Edinburgh, UK). From day 2 after infection, thin blood smears were taken. Mosquito blood feeds took place on day 4 pi, when all infected mice had sufficiently high gametocytaemia (a proportion of blood cells infected with gametocytes greater than 0.1%).

Blood feeds were carried out as described above. Ten cages, each containing approximately 100 *An. stephensi*, were used for *P. yoelii *infective feeds and the same numbers of uninfected mice were used for control feeds. Following the feeds, mosquitoes not taking a full blood meal were removed and half of the cages from both the *P. yoelii *and control groups were sprayed with the F52 formulation as detailed above. Sub-samples were then taken from these and from the unsprayed cages and divided into replicate cages for survival monitoring. The remaining mosquitoes were placed in one large cage per treatment for use on the gradients. This provided four treatments in total: 1) control uninfected, 2) *P. yoelii *infected, 3) F52 infected and 4) *P. yoelii *+ F52 infected. Gradient exposures were carried out in the same manner as described above from day 0 (the day of the feed) until day 4.

On day 8 after infection, 75 mosquitoes from each malaria treatment group (i.e. *P. yoelii *and *P. yoelii *+ F52 survivors from gradient- and non-gradient-exposed populations) were dissected to assess parasite presence and burden (number of oocysts per gut). Mosquitoes were dissected under a binocular microscope in 100 μl of M phosphate-buffered saline (PBS). After dissection the excised gut was covered with a cover slip and examined under a compound microscope for presence/absence of oocysts and the number of oocysts on infected midgets was counted. Infection was not monitored for the full sporogonic development cycle as the aim of the study was to determine thermal behaviour of the vector and with such short gradient exposure periods, no impact on Plasmodium was anticipated.

### Statistical analysis

*Anopheles stephensi *survival in both experiments was assessed by Kaplan-Meier survival analysis in SPSS for Windows v. 16, with differences in median survival times between treatments assessed using the Log rank test. Resting temperature on the gradient was analysed using GLM (SPSS v.16) with treatment, day of exposure and gradient included in the model. Oocyst prevalence in experiment 2 was analysed using a Pearson Chi-Square test. A negative binomial distribution was fitted to the oocyst intensity data (mean oocyst number per midgut of those infected) and any difference between fungal (i.e. *P. yoelii *+/- F52) and thermal regimes (i.e. exposed to gradient or maintained at constant temperature) tested by comparing the coefficients of the distributions (Generalized Linear Models function in SPSS v.16.).

**Figure 1:**
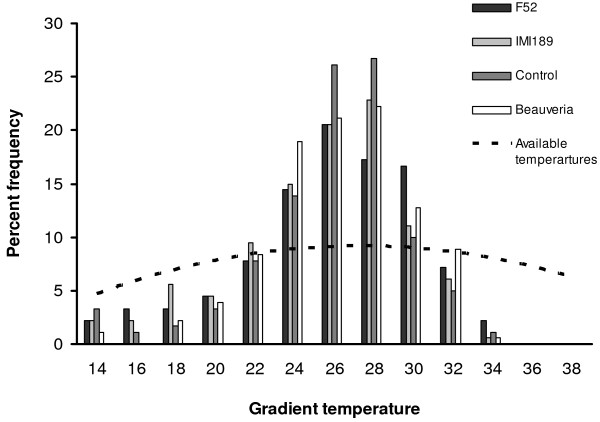
**Frequency distribution of fungi infected *An. stephensi *on a thermal gradient**. Percent frequency distribution of the pooled perching temperatures recorded from the thermal gradient for experiment 1. *Anopheles stephensi *were exposed to one of three fungal isolates or left untreated as a control. Dashed line shows the distribution of available temperatures in the gradient.

**Figure 2:**
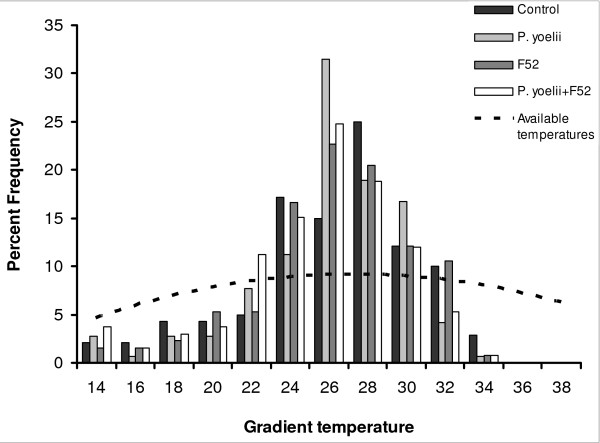
**Frequency distribution of fungus- and malaria-infected *An. stephensi *on a thermal gradient**. Percent frequency distribution of the pooled perching temperatures recorded from experiment 2. *Anopheles stephensi *were infected with the rodent malaria *P. yoelii*, the fungal isolate F52, a combination of the two or left untreated as a control. Dashed line shows the distribution of available temperatures in the gradient.

To test whether *Anopheles *showed any preference for a particular temperature or narrow range of temperatures, the actual perching temperatures were compared (each recorded perching temperature for all treatments pooled over the five days for experiment one and over the 4 days of experiment 2) with a distribution representing no temperature preference (see Figure 1 and Figure 2). To generate the no preference distribution the available temperatures on the surface and just beneath the lid of each channel was measured at 1 cm intervals. As noted above the available temperature range had a slightly non-linear distribution with tempertures available in the middle range (24–28°C) than at the cooler or hotter ends. The same number of data points was used as were recorded from each experiment (547 and 720 in experiment 1 and 2 respectively) and assigned them evenly according to the availability of temperatures recorded from the gradients. The two distributions were compared with a Kolmogorov-Smirnov non-parametric test.

## Results

### Experiment 1: *Anopheles stephensi *infected with fungal pathogens

#### Mosquito survival

Median survival time could only be computed for the *Beauveria *treatment (Median survival = 9.0 days, ± 0.24 days; 95% CI 8.42 – 9.48) as survival had not fallen below fifty percent in any other treatment (Mean percent survival at day 8 – Control = 98.7%; Beauveria = 38.0%; F52 = 79.3%; M189 = 99.3%). There was no difference in survival between control mosquitoes and those exposed to M189, but there was significantly faster mortality in both F52 (Log Rank Statistic (LRS) = 28.6, *P *< 0.001 and 31.2, *P *< 0.001, against Control and M189, respectively) and *Beauveria*-exposed insects (LRS = 129.1, *P *< 0.001 and 131.8, *P *< 0.001, against Control and M189, respectively). In addition *Beauveria*-infected mosquitoes had more rapid mortality compared with F52 (LRS = 48.8, *P *< 0.001).

#### Gradient resting temperature

**Figure 3:**
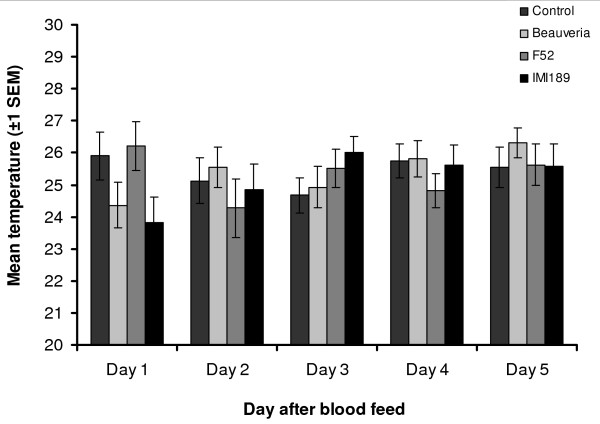
**Mean resting position of *An. stephensi *following infection with fungal pathogens**. Pooled daily mean perching temperature of *Anopheles stephensi *on a thermal gradient from experiment 1. Mosquitoes were treated with one of three entomopathogenic fungi (*Beauveria bassiana *isolate IMI391510, *Metarhizium anisopliae *var *anisopliae *isolate F52, or *M. anisopliae *var *acridum *isolate IMI330189) or left untreated as control

Daily mean resting temperatures for each treatment are shown in Figure 3 and the frequency distribution of resting temperatures for each treatment pooled across the five days in Figure 1. Overall mean (± s.e.m) resting temperature was 25.4 (± 0.28), 25.6 (± 0.27), 25.4 (± 0.33) and 24.9 (± 0.31) for Control, *Beauveria*, F52 and M189, respectively. There was no effect of exposure to fungal pathogens (F_3,119 _= 0.67 *P *> 0.5), or of time since infection (F_4,119 _= 0.53 *P *> 0.7) on the daily mean resting temperature of *An. stephensi*, nor any interaction between the two (F_12,119_, = 1.11 *P *= 0.36).

### Experiment 2: *Anopheles stephensi *infected with *P. yoelii*

#### Mosquito survival

Median survival times could not be computed for any treatment as none had survival less than fifty percent (Percent survival at day 8 – Control = 99.1%; *P. yoelii *= 98.0%; F52 = 84.1%; *P. yoelii *+ F52 = 86.2%). Both treatments exposed to F52 died at a faster rate than did control mosquitoes (LRS = 19.5, *P *< 0.001 for F52; LRS = 16.5, *P *< 0.001 for *P. yoelii *+ F52) or mosquitoes infected only with malaria parasites (LRS = 18.1, *P *< 0.001 for F52; LRS = 14.7, *P *< 0.001 for F52 + *P. yoelii*). There was no survival difference between *P. yoelii *infected and control mosquitoes (LRS = 0.72, *P *= 0.4), nor between F52 and *P. yoelii *+ F52 infected mosquitoes (LRS = 0.21, *P *= 0.65).

#### Gradient resting temperature

**Figure 4:**
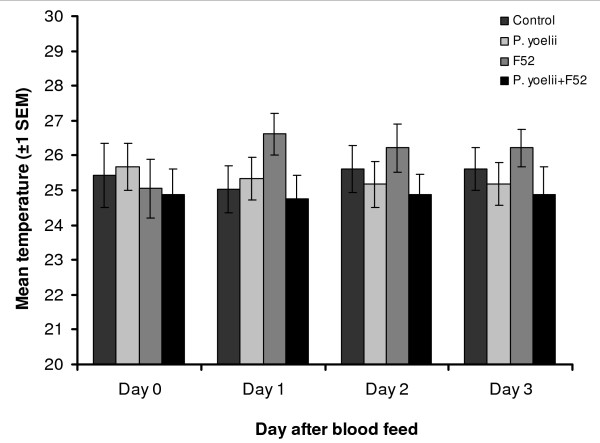
**Daily mean perching temperature of *Anopheles stephensi *infected with *P. yoelii***. Pooled daily mean perching temperature of *Anopheles stephensi *on a thermal gradient from experiment 2. Mosquitoes were infected with either the rodent malaria *P. yoelii *only, the *Metarhizium anisopliae *var *anisopliae *isolate F52 only, a combination of the two or left untreated as a control.

Daily mean resting temperatures are shown in Figure 4, with the frequency distribution of resting temperatures pooled across the four days for each treatment shown in Figure 2. Overall mean (± s.e.m) resting temperature was 25.6 (± 0.37), 25.5 (± 0.32), 25.7 (± 0.35) and 24.8 (± 0.35) for control, *P. yoelii*, F52 and *P. yoelii *+ F52, respectively. There was no significant difference in resting temperature between treatment groups on the gradient (F_2,95 _= 1.19, *P *> 0.3), nor was there an effect of time since infection (F_3,95 _= 0.24, *P *> 0.8), or interaction between treatment and time since infection (F_6,95 _= 0.89 *P *= 0.5) over the four day study period.

#### Malaria infection

**Figure 5:**
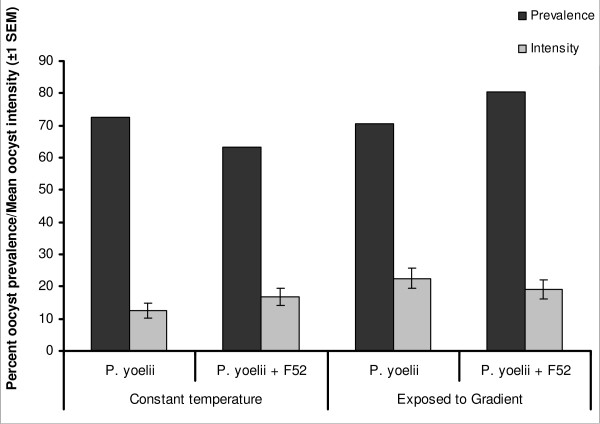
**Prevalence and mean intensity of *P. yoelii *infection in *An. stephensi***. Infection status of dissected *An. stephensi *on day 8 following blood meal. Dark grey bars indicated the percent of mosquitoes that had oocysts present on the midgut and light grey bars indicate the mean (± SEM) number of oocysts present. "Constant temperature" were those mosquitoes kept in the insectary at 26°C for the duration of the study and "Exposed to gradient" were those mosquitoes that had a daily 30 minute exposure to the gradient before being returned to the constant temperature environment.

Malaria prevalence and oocyst infection intensity are summarized in Figure 5 and Table 1. All malaria treatments showed similar oocyst prevalence (χ^2 ^= 1.13, *P *> 0.05). As expected, oocyst intensities followed a negative binomial distribution (Pearson Chi squared = 196.6, d.f. = 204, goodness of fit = 0.96), with mean and dispersion coefficients that did not differ between treatment group (Likelihood Ratio Chi-squared = 6.93, d.f. = 3, *P *> 0.05). Thus, there was no evidence that fungal or thermal regime affected prevalence of intensity of malaria infection.

**Table 1:** Prevalence and intensity of *P. yoelii *and fungal infection in *An. Stephensi*

	**Insects held at constant 26°C**		**Insects exposed to the gradient**	
	
	Prevalence	Oocyst burden (± SEM)	Prevalence	Oocyst burden (± SEM)
P. yoelii	72.4%	19.4 ± 3.20	70.7%	32.5 ± 7.78

P. yoelii + F52	63.2%	27.2 ± 5.53	80.3%	25.2 ± 3.67

Infection status of *An. stephensi *at day 8 post infection with *P. yoelii*. Mosquitoes were infected with *P. yoelii *only or *P. yoelii *and the fungal isolate F52 in combination. "Insects held at 26°C" were kept in the insectary throughout the study period whereas "Insects exposed to the gradient" were housed in the same insectary at all times apart from a 30 minute period every day where their thermal preference was determined on the gradient. "Prevalence" indicates the percent of mosquitoes that had a least one oocyst per midgut and "Oocyst burden" indicates the mean (± SEM) number of oocysts found per midgut.

### *Anopheles stephensi *resting temperature distribution

Examination of the frequency distribution of *An. stephensi *across both experiments (Figure 1 and Figure 2) suggested that the mosquitoes showed some temperature preference on the gradients. There was a significant difference between the perching temperatures (pooled for all treatments and all days) compared to the distribution expected if mosquitoes showed no preference across the available temperatures (Experiment 1: Kolmogorov-Smirnov test z = 4.24, *P *< 0.01: observed mean: 25.3 ± 0.15°C, expected under no preference: 26.0 ± 0.26°C; Experiment 2: Kolmogorov-Smirnov test z = 3.66, *P *< 0.01: observed mean – 25.4 ± 0.17°C, expectation under no preference: 26.0 ± 0.29°C). In particular, mosquitoes avoided the hottest temperature available and the coldest (Figure 1 and Figure 2)

## Discussion

None of the three fungal isolates used in this study elicited a change in thermal behaviour of *An. stephensi*. Unlike a number of other insect-fungus studies (including some with one of the isolates used [11]), fungal infection did not cause mosquitoes to seek higher resting temperatures (a hyperthermic, fever response), nor did it result in a decrease in chosen resting temperatures (a hypothermic response) [e.g. [40]]. This absence of altered thermal behaviour is unlikely to be due to failure to initiate infection: both *Beauveria*- and F52-exposed insects had decreased survival compared with control insects. For M189 there was no effect on *An. stephensi *survival during the course of the investigation but previous studies have shown this isolate to only cause increased mortality rates after 20 days (unpublished data), which is beyond the monitoring period of the current study. Given the delivery system used, there is no reason to suspect that mosquitoes escaped infection.

Adding malaria to the mix did not alter this result. *An. stephensi *infected with *P. yoelii *alone, or in combination with fungal isolate F52, chose to rest at the temperatures chosen by the relevant controls. There was no evidence for selection of warmer temperatures which might enhance rates of parasite development after invasion (but note thermal behaviour was only monitored for the earlier part of the incubation period). Similarly, there was no evidence for manipulation of resting temperature by the parasite to avoid exposure to damaging temperatures at the initial post blood feeding stage.

It is not clear how the use of an artificial laboratory model of malaria may differ from *Plasmodium falciparum *infections of *Anopheles *species in the field [41]. Some studies using a rodent model system have yielded useful insights into *Plasmodium*-vector interactions. For example, studies on *P. yoelii *manipulation of *An. stephensi *fecundity have been mirrored in field studies on *P. falciparum *infection of *An. gambiae *[42,43]. The experiments reported here would seem well suited to an initial investigation with a model system before being extended to a natural system.

Mosquitoes did avoid the hottest temperatures in both experiments. Of the recorded resting temperatures, 24–36% were at or above 28°C, a temperature which impacts on early stage malaria development [32-34]. Yet malaria-infected mosquitoes did not exploit these temperatures. These results provide no evidence that the parasite is able to manipulate host-resting behaviour to avoid detrimental micro-environments or the vector to exploit them. In part, mosquito behaviour might already result in avoidance of the hottest niches (e.g. light intensity is a key factor in *Anopheles *resting site selection [44] and will lead to avoidance of sun patches) and conditions at common resting sites might generally be benign. There are very few data on the temperature of outdoor resting sites and few studies routinely measure the thermal environment inside domestic dwellings. Where this has been carried out, temperatures tend to exceed 28°C for just short periods [45,46] and only a combination of exposed settings and dry season conditions see temperatures inside huts consistently exceed 30°C [47].

For both the fungal and malaria infections, the apparent lack of thermal response is unlikely to be due to a failure by mosquitoes to detect infection. Entomopathogenic fungi produce an array of secondary metabolites on entry into the insect haemocoel that can disrupt haemocytic responses to the invading pathogen [48,49]. In other insects, these metabolites stimulate behavioural fever [e.g. [38]]. Similarly, *Anopheles *can recognize and respond to malaria infection as the ookinetes invade and cross the midgut [50-52]. The time course of ookinete development and midgut invasion is temperature dependent but is almost certainly captured under the conditions of the current study [32,50].

From previous studies, insect species that exhibit altered thermal selection in response to infection tend to be active thermoregulators and/or relatively large bodied [e.g. [11,26,53-55]], rather than small bodied [39,56]. The apparent influence of body size is consistent with the fact that small-bodied organisms have a high surface to volume ratio, and the costs of maintaining body temperatures at a particular set point, and certainly a higher set point, are thought to outweigh any potential benefit [57]. The absence of marked hypo/hyperthermic behavioural responses observed in *An. stephensi *is consistent with this. What is noteworthy, however, is that in both experiments, the distribution of mosquitoes on the thermal gradients was markedly different from a distribution achieved if there had been no temperature preference (Figure 1 and Figure 2). Avoidance of desiccating temperatures [21] and sub-operative temperatures at either extreme of the gradient may account for a narrower distribution in-part. However, the frequency distribution of resting positions was surprisingly tight: 44% and 45% of insects in the two experiments rested at temperatures between 24–27°C. This indicates some capacity of *An. stephensi *to behaviourally thermoregulate. Adaptation to a change in the thermal environment experienced by an organism has been well documented [e.g. [58-60]] and the lower resting temperatures recorded here in comparison to the temperatures representing development rate maxima [18] may simply reflect acclimation of a laboratory colony reared at 26–27°C for more than 10 years. Conversely the clumping of resting temperatures may reflect a preference for resting sites where body temperature is optimized for other physiological processes (e.g. feeding and digestion) which may have a lower optima than that of the maximal rate of development [61,62].

The absence of altered thermal behaviour in response to fungal or malarial infection does not mean, of course, that environmental temperatures are irrelevant for either host-parasite interaction. The growth of a fungus, the extrinsic incubation period of malaria, and various aspects of mosquito life history (such as duration of gonotrophic cycle, adult longevity), are all temperature dependent. Even subtle non-linearities between the temperature responses of the three interacting organisms could lead to markedly different impacts of a biopesticide on malaria transmission, depending on local environmental conditions ([see also [13]]). Understanding these potentially complex temperature-dependent interactions is a key area for further research, both for development of novel biopesticide interventions and, in the case of *Plasmodium *development to fully understand the geographic and temporal variation in malaria risk, and the implications of climate change. In the meantime, and with the usual cautions about generalizing from laboratory experiments with animal models, it can be concluded that, in contrast to locust biopesticides, there is no evidence that thermal behaviour by *Anopheles *will compromise the efficacy of fungal biopesticides for malaria control.

## Competing interests

The authors declare that they have no competing interests.

## Authors' contributions

SB designed and conducted the experimental work, analyzed and summarized the data and drafted the manuscript. MT and AR co-supervised the experimental work and contributed to the manuscript.
